# Alcohol consumption associated with suicidal ideation, and suicide attempts in substance users: A cross-sectional study of an addiction registry in western Iran

**DOI:** 10.1371/journal.pone.0317456

**Published:** 2025-02-04

**Authors:** Vahid Farnia, Mahsa Mohebian, Omran Davarinejad, Denise A. Hien, Safora Salemi, Sara Hookari, Hafez Bajoghli, Bahareh Rahami

**Affiliations:** 1 Substance Abuse Prevention Research Center, Health Institute, Kermanshah University of Medical Sciences, Kermanshah, Iran; 2 Department of Psychiatry, School of Clinical Sciences, Faculty of Medicine, Nursing and Health Sciences, Monash University, Clayton, VIC, Australia; 3 Clinical Research Development Center, Imam Khomeini and Mohammad Kermanshahi and Farabi Hospitals, Kermanshah University of Medical Sciences, Kermanshah, Iran; 4 Center of Alcohol and Substance Use Studies, Rutgers University-New Brunswick, Piscataway, New Jersey, United States of America; 5 Iranian National Center for Addiction Studies (INCAS), Tehran University of Medical Sciences, Tehran, Iran; University of Botswana, BOTSWANA

## Abstract

**Background:**

Suicide is recognized as a major problem worldwide and is particularly prevalent among specific groups, including individuals with substance use disorders (SUD_s_). The present study aimed to investigate alcohol consumption as a risk factor for suicidal ideation and attempts among those with substance use disorders (SUDs) in western Iran.

**Methods:**

This is a cross-sectional study, involving 1,112 individuals with SUDs who sought treatment at Farabi Hospital in Kermanshah, Iran, between the years 2019 and 2021. These participants were included in the study through a convenient sampling method as part of an interview-based assessment study.

**Results:**

The participant’s average age was 37.97 years, and 982 were male (94.7%). Overall, 285 (27.5%) individuals had a history of suicide attempts, 316 (30.5%) individuals reported suicidal ideation, and 463 (41.6%) were alcohol users. In individuals who consumed alcohol, the prevalence of suicidal ideation (172 (37.2%) individuals), and a history of suicide attempts (156 (33.8%) individuals) was significantly higher compared to non-alcohol users. There was a statistically significant relationship between alcohol consumption and a history of suicide attempts (p < 0.05). The probability of suicide attempted in people with a history of alcohol consumption was 1.5 times, and in patients with a history of simultaneous substance use, it was 1.4 times that of other patients (all Ps < 0.05).

**Conclusion:**

Our study results revealed that alcohol consumption among individuals with SUD_s_ is associated with increased rates of suicidal ideation, attempts, and death. Therefore, clinicians should consider it as a separate suicide risk factor.

## Introduction

Suicide is one of the social problems that leads to the loss of active members of society and is considered a significant mental health problem. It is among the top 20 leading causes of death worldwide for all age groups [1], the third leading cause of death among individuals aged 15 to 44, and the second leading cause of death among individuals aged 10 to 24 [2]. Suicide is one of the largest contributors to premature mortality [3]. The number of individuals who die due to homicide and suicide is much higher than those who lose their lives in warfare. In fact, for every death caused by war, there are three deaths due to homicide, but five deaths due to suicide [2]. Approximately one million people are estimated to lose their lives annually due to suicide, which means the global mortality rate is 16 per 100,000 or one death every 40 seconds [2].

Suicide is forbidden in Islamic cultures. Moreover, the exact rate of attempted suicide may be underestimated because of the religious or cultural stigma and criminal offenses. Twenty Islamic countries may punish suicide attempters with imprisonment, fine, or both, as well as other social deprivations [4]. Yet, there are no accurate statistics about the prevalence of suicide and suicide attempts in Iran. In a systematic review in Iran [5], the prevalence of suicide attempts in the whole population was 131.0 per 100,000 people (152 per 100,000 women and 128 per 100,000 men). Moreover, the prevalence of suicide death was 8.14 per 100,000 people in the general population (5.0 per 100,000 women and 9.1 per 100,000 men).

A multitude of factors can contribute to an increased risk of suicide among individuals with substance use disorders (SUD_s_). These risk factors encompass various levels, ranging from the lowest to the highest levels, including individual, familial, population-related, sociocultural, and daily stress factors. Each of these risk factors can serve as a contributing factor to increased suicidal tendencies and completed suicide. One such factor is a history of alcohol consumption. Systematic investigations demonstrate that alcohol use disorders can pose a risk for suicide [6]. Persistent alcohol consumption is a global world health issue that comes with serious individual and societal consequences. In addition to chronic diseases that may develop in individuals who consume excessive amounts of alcohol over a prolonged period, a significant portion of the disease burden arises from intentional and unintentional injuries, such as violent behaviors, suicide, and traffic accidents. Alcohol use disorders rank as the second most common psychiatric disorder, following depression, among individuals who attempt suicide [7,8]. On a global scale, alcohol consumption is implicated in approximately 3.3 million deaths per year (accounting for 5.9% of all deaths), and nearly 4.6% of years lived with disability are attributed to alcohol consumption [9,10].

Alcohol consumption is forbidden in Iran. Individuals caught consuming alcohol can be punished by lashes, and, in the case of multiple offenses, death. Furthermore, the prevalence of alcohol consumption in Iran cannot be estimated because large variations have been observed in the reported prevalence of alcohol consumption [11]. The prevalence of alcohol consumption in Iran varied from 0.03% to 68.0% in different regions, 0.3% to 66.6% among males and 0.2% to 21.0% among females [12].

Isaacs et al. [13] demonstrated in a meta-analysis that alcohol consumption was associated with a 94% increased risk of suicide. Their study highlights the use of alcohol as a significant risk factor for suicide and emphasizes the importance of monitoring alcohol consumption in individuals at risk of suicide and screening for suicide in alcohol users. Lange et al. [14], in a study conducted in the United States between 2003 and 2018, showed an increased prevalence of alcohol consumption before suicide among women. DeCou and Skewes [15] also found that alcohol consumption can predict suicide attempts, indicating a complex relationship between alcohol and psychiatric disorders. Alcohol consumption can have negative effects on mental health, leading to mental disorders and an increased risk of suicide [16,17]. Countries with higher alcohol consumption rates generally have higher suicide rates. Additionally, current evidence demonstrates a reactive relationship between alcohol dependence and suicide attempts [18,19]. Considering the aforementioned points, suicide is a recognized global issue and a major public health problem.

Younger age of individuals attempting suicide further compound the challenges. Furthermore, suicide negatively affects the mental health of family members of the victims, impairs their social life, and increases the likelihood of its occurrence within the family and society. It seems that the first step in social planning to prevent suicide is to identify its associated factors. Moreover, suicide can be a significant public health concern, and considering the link between SUD_s_ and suicide, understanding the nature and mechanism of the relationship between these two factors is crucial for designing and developing appropriate interventions. Alcohol consumption is illegal in Iran and due to cultural and religious beliefs, thus the available population-based research findings on alcohol consumption are inadequate. Additionally, although alcohol consumption is a risk factor for suicidal behavior, the nature of the relationship is unclear. Therefore, this study was conducted to identify factors that affect suicidal ideation and suicide attempts among Iranian individuals with SUDs, both with and without a history of alcohol consumption.

## Materials and methods

In a cross-sectional study, the inpatients of Farabi Hospital (Kermanshah University of Medical Sciences, Kermanshah, Iran) with substance use disorders (SUD_s_) that were registered in the registry of addiction disorders in 2019–2021 participated.

Demographic information, self-reported histories, medical records, clinical data, and relevant information regarding substance use, alcohol consumption, suicide attempts and suicidal ideation were recorded, which is being implemented and registered at Farabi Hospital in Kermanshah. 1,112 patients who were diagnosed with SUD_S_ in the Addiction Disorders Registry Project at Farabi Hospital based on the inclusion and exclusion criteria were selected using census sampling.

Inclusion criteria were as follows: (1) Registration in the registry of addiction disorders (2) diagnosis of SUDs, based on DSM-5 diagnostic criteria plus TLC (Thin Layer Chromatography) test result and as ascertained by a trained and experienced psychiatrist; (3) willing and able to comply with the study conditions; (4) signed written informed consent. Exclusion criteria were: (1) severe psychiatric comorbidities, including intellectual disability (IQ < 80, as ascertained by the Wechsler IQ test); (3) withdrawal from the study. All information in the present study was obtained through interviews conducted by an experienced clinical psychologist.

A demographic questionnaire and clinical checklist included gender (male; female), age (years), Type of Referral (outpatient; hospitalized), methods of referral (self-referral; family coercion; referral from an organization or institution), education (illiterate; primary school; high school; diploma; associate degree; bachelor’s degree and higher), job (currently studying independent; self-employed; governmental; unemployed), marital status (single; married; divorced; widowed), divorce experience related to addiction (yes, no), monthly economic income of the individual (10 million > ; ≥ million10 rial), housing status (father’s house; rented house; personal house; living in a camp), birth order (number), first age of substance use (years), first substance used (opium, stimulant, hallucinogens, alcohol, sedative drugs),duration of substance-use in months (months), history of simultaneous substance use (yes, no), dose of alcohol consumed (CC), purity percentage of alcohol consumed (%), family history of alcohol consumption (yes, no), history of alcohol cessation attempts (yes, no), history of legal and judicial problems in the family (yes, no), history of psychiatric disorders in the family (yes, no), history of psychiatric hospitalization in the family (yes, no), history of suicidal ideation(lifetime) (yes, no), frequency of suicidal ideation (number), history of suicide attempts (yes, no), number of suicide attempts (number), methods of suicide (poisoning, hanging, weapon, causing an accident, self-immolation, electrocution, air injection), history of suicide related to addiction in an individual (yes, no, unknown), history of suicide during substance use (yes, no, unknown), history of suicide during substance cessation (yes, no, unknown), having a conversation with someone during a suicide attempt (yes, no, unknown), alcohol consumption (lifetime).

A criminal offence is an act or an omission that is unlawful and punishable by the specific laws of a country. One definition of a criminal offence is an act that is forbidden as it is harmful to an individual, individuals, a community, a society or the state, and is therefore punishable by law. Although there are many different kinds of criminal offence, criminal acts can generally be divided into five primary categories: crimes against a person, crimes against property, inchoate crimes, statutory crimes, and financial crimes.

After obtaining permission and making relevant arrangements with those involved in the Addictive Disorders Registry and obtaining a code of ethics from the Ethics Committee of Kermanshah University of Medical Sciences, the researchers conducted clinical interviews and studied the medical records of eligible participants in the study. It should be noted that patient information was kept confidential without disclosing any names, and the researcher committed to maintaining the confidentiality of all the collected data.

### Statistical analysis

Subsequently, the gathered information, including demographic data (age, gender, marital status, education, occupation, economic status, etc.), self-reported histories (medical history, etc.), medical records (physical and psychiatric disorders), and substance use histories (type of substances used, age of first substance use, family history of addiction or substance use, etc.), were entered into the SPSS-20 software by a statistician. Finally, appropriate analytical methods were employed to perform statistical analysis, and relevant graphs and tables were designed to present the findings.

To examine the relationship between factors categorized with alcohol consumption, the Chi-Square Test was used. The Fisher Exact Test was employed to adjust the Chi-Square Test if necessary. Multivariable logistic regression methods were utilized to determine the factors that contribute to suicidal attempts in mental patients. The nominal level of significance was set at alpha < 0.05. All statistical computations were performed with SPSS^®^ 20.0 (IBM Corporation, Armonk, NY, USA) for Apple Mac®.

### Ethical considerations

Ethical considerations were taken into account in this study so that the study objectives were clearly explained in a comprehensible manner to the participants. Participation in the research was entirely voluntary and without any form of coercion. Participants were assured that the results of the study and any tests conducted would be kept strictly confidential, and confidentiality was maintained by the principle of privacy. Participants’ personal identity information was not recorded, and only codes provided by the participants were used for data identification. Participants had the right to withdraw from the research at any stage, including data collection. Informed consent was obtained from all participants, and they signed the consent form. This study was registered at the Kermanshah University of Medical Sciences in Iran and obtained ethical approval from the university’s ethics committee.) IR.KUMS.REC.1397.120).

## Results

As displayed in Table 1, the average age of the participants was 38.0 years (94.7% males, and 5.3% females). Among 1,112 individuals with Substance Use Disorders (SUDs), 575 individuals (51.7%) reported no alcohol consumption, while 463 individuals (41.6%) had a history of alcohol consumption. Population ratios indicated that the proportion of non-drinking women (8.5%) was more than six times higher than that of women who consumed alcohol (1.3%). Among men, 457 (98.7%) were alcohol users. Additionally, more than half of the alcohol users were under the age of 35, whereas in the non-users group, more than half were above 35. Among the participants, 992 individuals (95.7%) had a history of psychiatric hospitalization, while 45 individuals (4.3%) sought outpatient care, indicating that the proportion of individuals seeking outpatient care among non-users (7.1%) was more than seven times higher than that among users (0.9%).

**Table 1:** Distribution of demographic characteristics based on alcohol consumption history in study participants.

		Category	Alcohol consumption Number (%)		Total	Chi-square statistics (P-Value)
			No	Yes		
Demographic Characteristics	Current Age Category	<25	39 (6.8)	49 (10.6)	88 (8.5)	58.44*** **(<0.0001)**
		26–35	146 (25.4)	194 (41.9)	340 (32.8)	
		36–45	223 (38.9)	162 (35.0)	385 (37.1)	
		>45	166 (28.9)	58 (12.5)	224 (21.6)	
	Gender	Female	49 (8.5)	6 (1.3)	55 (5.3)	26.75*** **(<0.0001)**
		Male	525 (91.5)	457 (98.7)	982 (94.7)	
	Type of Referral	Outpatient	41 (7.1)	4 (0.9)	45 (4.3)	24.34*** **(<0.0001)**
		Hospitalized	533 (92.9)	459 (99.1)	992 (95.7)	
	Method of Referral	Self-referral	496 (86.4)	368 (79.5)	864 (83.3)	8.97* **(0.011)**
		Family coercion	64 (11.1)	76 (16.4)	140 (13.5)	
		Referral from an organization or institution	14 (2.4)	19 (4.1)	33 (3.2)	
	Education	Illiterate	42 (7.3)	16 (3.5)	58 (5.6)	16.52* **(0.011)**
		Primary school	108 (18.8)	65 (14.0)	173 (16.7)	
		Middle school	198 (34.5)	200 (43.2)	398 (38.4)	
		High school	167 (29.1)	136 (29.4)	303 (29.2)	
		Diploma	25 (4.4)	15 (1.4)	40 (3.9)	
		Associate degree	25 (4.4)	22 (4.8)	47 (4.5)	
		Bachelor’s degree and higher	9 (0.9)	9 (0.9)	18 (1.7)	
	Job	Currently Studying Independent	3 (0.5)	3 (0.6)	6 (0.6)	14.60** **(0.002)**
		Self-employed	360 (62.7)	338 (73.0)	698 (67.3)	
		Governmental	54 (9.4)	23 (5.0)	77 (7.4)	
		Unemployed	157 (27.4)	99 (21.4)	256 (24.7)	
	Marital Status	Single	175 (30.5)	226 (48.8)	401 (38.7)	51.05** **(<0.0001)**
		Married	329 (57.3)	166 (35.9)	495 (47.7)	
		Divorced	64 (11.1)	69 (14.9)	133 (12.8)	
		Deceased spouse	6 (1.0)	2 (0.4)	8 (0.8)	
	Divorce Experience Related to Addiction	No	43 (50.9)	36 (49.3)	79 (50.0)	**0.025 (0.500)**
		Yes	42 (49.4)	37 (50.7)	79 (50.0)	
	Monthly Economic Income of the Individual (In ten million Iranian Rials)	<2	147 (49.5)	141 (50.4)	288 (49.9)	**2.85 (0.538)**
		2–4	92 (31.0)	82 (29.3)	174 (30.2)	
		4–6	38 (12.8)	39 (13.9)	77 (13.3)	
		6–8	14 (4.7)	8 (2.9)	22 (3.8)	
		10–8	6 (2.0)	10 (3.6)	16 (2.8)	
	Housing Status	Father’s house	222 (38.7)	254 (54.9)	476 (45.9)	40.74*** **(<0.0001)**
		Rented house	172 (30.0)	116 (25.1)	288 (27.8)	
		Personal house	176 (30.7)	81 (17.5)	257 (24.8)	
		Living in a camp	4 (0.7)	12 (2.6)	16 (1.5)	
	Birth order	First child	152 (26.4)	126 (27.3)	278 (26.8)	**8.072 (0.326)**
		Second child	100 (17.4)	79 (17.1)	179 (17.3)	
		Third child	95 (16.5)	74 (16.0)	169 (16.3)	
		Fourth child	80 (13.9)	63 (13.6)	143 (13.8)	
		Fifth child	41 (7.1)	53 (11.5)	94 (9.1)	
		Sixth child	37 (6.4)	24 (5.2)	61 (5.9)	
		Seventh child	30 (5.2)	18 (3.9)	48 (4.6)	
		Eighth child	40 (7.0)	25 (5.4)	65 (6.3)	

The proportion of individuals who sought treatment with family coercion or through organization referrals was higher among alcohol users than non-users. The proportion of individuals with high school or university education was also higher among alcohol-consuming patients compared to non-users. Furthermore, the proportion of unemployed individuals or those with government jobs or high school or university education was higher among non-consuming patients compared to users.

Among alcohol users, the proportion of individuals with a history of divorce (50.7%) was slightly higher than that among non-users (49.4%). Additionally, the proportion of individuals residing in camps among alcohol users (0.7%) was more than three times higher than that among non-users (0.2%). In both groups, living in the father’s house was reported with the highest frequency. This was while the ratio of reporting living in the camp for alcohol consuming patients 12 (2.6%) was significantly higher than non-alcohol consuming individuals 4 (0.7%).

Furthermore, the correlation analysis showed a strong significant relationship between alcohol consumption history and current age group, gender, type, and mode of referral, education level, occupation, marital status, and housing status (all p < 0.05) (Table 1).

Table 2 present results of the comparison between those with alcohol consumption and those without on substance use characteristics. Investigation of substance use histories in these patients showed that the ratio of patients who reported initiating substance use before the age of 15 was more than twice as high in the group of patients with a history of alcohol consumption (104, 22.5%) compared to those without alcohol consumption history (61,10.6%). Individuals with a history of alcohol consumption-initiated substance use at younger ages. Additionally, individuals with a history of alcohol consumption reported a shorter duration from the onset of substance use. Simultaneous use of multiple substances was also reported more frequently in the group with an alcohol consumption history of (130, 28.1%) compared to the group without alcohol consumption of (95, 16.5%). The average alcohol dose consumed by patients was reported as (1,040.4 mL, 607.9 cc), and the percentage of alcohol used was reported as (21.41%, 54.2%). Nearly 25% of the patients reported an alcohol percentage above 65%. Among the alcohol users, 124 individuals (26.8%) had a history of alcohol consumption in their families. Among alcohol users, 395 individuals (85.5%) reported a history of attempting to quit alcohol consumption. The use of opium as the first substance was nearly one and a half times more prevalent in the group of individuals without an alcohol consumption history (59.4%, 341) compared to those with alcohol consumption history (175, 37.8%).

**Table 2:** Distribution of substance use characteristics based on alcohol consumption history in the study participants.

		Category	Alcohol consumption Number (%)		Total	Chi-square Statistics (P-Value)
			No	Yes		
Substance Use History	First Age of Substance Use	<=15	61 (10.6)	104 (22.5)	165 (15.9)	88.31*** **(<0.0001)**
		16–25	316 (55.0)	305 (65.9)	621 (59.8)	
		26–35	130 (22.6)	46 (9.9)	176 (17.0)	
		36–45	46 (8.0)	7 (1.5)	53 (5.1)	
		>45	22 (3.8)	1 (0.2)	23 (2.2)	
	First Substance Used	Alcohol	0 (0.0)	39 (8.2)	39 (3.8)	117.81*** **(0.0001)**
		Opium	341 (59.4)	175 (37.8)	516 (49.7)	
		Opium juice (Shirah)	39 (6.9)	36 (7.8)	75 (7.2)	
		Heroin	43 (7.5)	26 (5.6)	69 (6.6)	
		Morphine	1 (0.2)	0 (0.0)	1 (0.1)	
		Codeine	0 (0.0)	2 (0.4)	2 (0.2)	
		Methadone	20 (3.5)	12 (2.6)	32 (3.1)	
		Buprenorphine	1 (0.2)	0 (0.0)	1 (0.1)	
		Tramadol	47 (8.2)	64 (13.8)	111 (10.7)	
		Crack	2 (0.3)	6 (1.3)	8 (0.8)	
		Cocaine	2 (0.3)	0 (0.0)	2 (0.2)	
		Methamphetamine	31 (5.4)	25 (5.4)	56 (5.4)	
		Cannabis	37 (6.4)	76 (16.4)	113 (10.9)	
		Ecstasy	0 (0.0)	1 (0.2)	1 (0.1)	
		Sedative drugs	2 (0.3)	2 (0.4)	4 (0.4)	
		Tears	6 (1.0)	0 (0.0)	6 (0.6)	
		Temazepam	2 (0.3)	0 (0.0)	2 (0.2)	
	Duration of substance-use in Months	0–24	181 (31.5)	163 (35.2)	344 (33.1)	**4.884 (0.299)**
		48–24	93 (16.2)	82 (17.7)	175 (16.9)	
		72–43	60 (10.4)	55 (11.9)	115 (11.1)	
		96–73	44 (7.7)	30 (6.5)	74 (7.1)	
		>96	197 (34.3)	133 (28.7)	330 (31.8)	
	History of simultaneous substance use	No	480 (83.5)	333 (71.9)	813 (78.3)	20.17*** **(<0.0001)**
		Yes	95 (16.5)	130 (28.1)	225 (21.7)	
	Dose of alcohol consumed (CC)	0–500	0 (0.0)	333 (71.9)	333 (71.9)	**----**
		5,000–1,000	0 (0.0)	99 (21.4)	99 (21.4)	
		>1,000	0 (0.0)	31 (6.7)	31 (6.7)	
	Purity percentage of alcohol consumed	0–35%	0 (0.0)	27 (5.8)	27 (5.8)	**-----**
		36–65%	0 (0.0)	324 (70.0)	324 (70.0)	
		66–100%	0 (0.0)	112 (24.2)	112 (24.2)	
	Family history of alcohol consumption	No	1 (100.0)	338 (73.2)	339 (73.2)	**0.367 (0.732)**
		Yes	0 (0.0)	124 (26.8)	124 (26.8)	
	History of alcohol cessation attempts	No	0 (0.0)	68 (14.7)	68 (14.7)	**----**
		Yes	0 (0.0)	395 (85.5)	395 (85.5)	

* is significant at the 0.05% level. ** is significant at the 0.01% level. *** is significant at the 0.001% level.

For the alcohol consuming group, the ratio of reported duration of drug use in all time categories less than 72 months, was more than the non-drinking group. This was despite the fact that for the duration of drug consumption of more than 72 months, the ratio reported for alcohol consuming patients was lower than the non-consuming group. The dose of alcohol used for the majority of alcohol users 333 (71.9%) was 0–500 cc and for 31 (6.7%) patients it was more than 1,000 cc. Also, the purity of the percentage of consumed alcohol was reported for the vast majority of consumers 324 (70.0%) of patients, 36–65% and for 112 (24.2%) patients, 66–100%. In the alcohol consuming group, 124 (26.8%) patients reported a positive family history of alcohol consumption and 395 (85.5%) reported a history of taking action to quit alcohol consumption. Correlation results showed that there was a statistically significant relationship between the studied groups with the age of first consumption, the first substance consumed, and the history of simultaneous consumption of several substances (all p < 0.001).

The examination of family histories of the patients showed that the presence of a history of legal and judicial problems in the families of alcohol users (48.2%, 223) was nearly double that of individuals without alcohol consumption history (29.3%, 168), and this relationship was statistically significant (p < 0.05). The presence of psychiatric disorders and a history of psychiatric hospitalization in the family were slightly higher in the group of alcohol users compared to non-users, but this relationship did not reach statistical significance (all ps > 0.05). The results of these investigations are presented in Table 3.

**Table 3:** Distribution of family history among study participants based on alcohol consumption.

		Category	Alcohol consumption Number (%)		Total	Chi-square Statistics (P-Value)
			No	Yes		
Family History	History of legal and judicial problems in the family	No	406 (70.7)	240 (51.8)	646 (62.3)	38.96*** **(<0.0001)**
		Yes	168 (29.3)	223 (48.2)	391 (37.7)	
	History of psychiatric disorders in the family	No	524 (91.1)	420 (90.9)	944 (91.0)	**0.015 (0.493)**
		Yes	51 (8.9)	42 (9.1)	93 (9.0)	
	History of psychiatric hospitalization in the family	No	532 (92.5)	419 (91.1)	951 (91.7)	**0.729 (0.696)**
		Yes	42 (7.3)	41 (8.9)	83 (8.3)	

* is significant at the 0.05% level. ** is significant at the 0.01% level. *** is significant at the 0.001% level.

The examination of the distribution of suicide histories among the patients under study showed that over 30% of the patients had experienced suicidal ideation with an average of 2.49 occurrences (1.51 occurrences on average). The prevalence of suicidal ideation was significantly higher in alcohol users (37.2%, 172 individuals) compared to non-users (25.1%, 144 individuals) (p < 0.05). The number of occurrences of suicidal ideation was also higher in alcohol users, although this difference did not reach statistical significance (p > 0.05). Further analyses revealed that 27.5% (285 individuals) had a history of suicide attempts with an average of 2.43 attempts (1.51 attempts on average). A history of suicide attempts was significantly more prevalent in alcohol users (33.8%, 156 individuals) compared to non-users (22.5%, 129 individuals) (p < 0.05). The number of suicide attempts was also higher in alcohol users, with 44.3% (69 individuals) reporting more than two suicide attempts compared to 30.2% (39 individuals) in the non-user group (p > 0.05).

The most common methods of suicide attempts were poisoning, the use of weapons, and hanging, observed in both groups with the highest frequency. A history of suicide was also found to be more prevalent among individuals with alcohol use disorders in the alcohol user group (16.5%, 76 individuals) compared to individuals without alcohol consumption (10.1%, 58 individuals). The history of suicide during substance use was significantly higher in the alcohol user group (25.5%, 118 individuals) compared to those without alcohol use (18.5%, 106 individuals). This relationship also held for the history of suicide during substance cessation, with 8.0% (37 individuals) in the alcohol-users group and 4.5% (26 individuals) in the non-user group having a history of suicide attempts during substance cessation. Significant differences were reported in discussing the decision to commit suicide with others in alcohol users compared to non-users (all ps < 0.05). The results of these investigations are presented in Table 4.

**Table 4:** Distribution of suicide history in patients based on alcohol consumption history.

		Category	Alcohol consumption (%)Number		Total	Chi-square Statistics (P-Value)
			No	Yes		
Patients’ suicide histories	History of suicidal ideation	No	429 (74.9)	290 (62.8)	719 (69.5)	17.65** **(<0.0001)**
		Yes	144 (25.1)	172 (37.2)	316 (30.5)	
	Frequency of suicidal ideation	1	62 (43.1)	56 (32.6)	118 (37.3)	**5.94 (0.204)**
		2	34 (23.6)	39 (22.7)	73 (23.1)	
		3	18 (12.5)	23 (13.4)	41 (13.0)	
		4	10 (6.9)	15 (8.7)	25 (7.9)	
		5	20 (13.9)	39 (22.7)	59 (18.7)	
	History of suicide attempts	No	444 (77.5)	306 (66.2)	750 (72.5)	16.33*** **(<0.0001)**
		Yes	129 (22.5)	156 (33.8)	285 (27.5)	
	Number of suicide attempts	1	59 (45.7)	53 (34.0)	112 (39.3)	**6.989 (0.136)**
		2	31 (24.0)	34 (21.8)	65 (22.8)	
		3	15 (11.6)	22 (14.1)	37 (13.0)	
		4	7 (5.4)	11 (7.1)	18 (6.3)	
		5	17 (13.2)	36 (23.1)	53 (18.6)	
	Methods of suicide	Poisoning	82	97	179 (100.0)	**-----**
		Hanging	27	43	70 (100.0)	
		Weapon	39	61	100 (100.0)	
		Causing an accident	4	4	8 (100.0)	
		Self-immolation	5	4	9 (100.0)	
		Electrocution	2	3	5 (100.0)	
		Air injection	4	0	4 (100.0)	
	History of suicide related to addiction in an individual	No	491 (85.7)	385 (83.3)	876 (84.6)	24.78*** **(<0.0001)**
		Yes	58 (10.1)	76 (16.5)	134 (12.9)	
		Unknown	24 (4.2)	1 (0.2)	25 (2.4)	
	History of Suicide during substance use	No	442 (77.1)	343 (74.2)	785 (75.8)	23.65*** **(<0.0001)**
		Yes	106 (18.5)	118 (25.5)	224 (21.6)	
		Unknown	25 (4.4)	1 (0.2)	26 (2.5)	
	History of suicide during substance cessation	No	522 (91.1)	423 (91.6)	945 (91.3)	20.21*** **(<0.0001)**
		Yes	26 (4.5)	37 (8.0)	63 (6.1)	
		Unknown	25 (4.4)	2 (0.4)	27 (2.6)	
	Having a conversation with someone during a suicide attempt	No	548 (95.6)	444 (96.1)	992 (95.8)	18.21*** **(<0.0001)**
		Yes	9 (1.6)	18 (3.9)	27 (2.6)	
		Unknown	16 (2.8)	0 (0.0)	16 (1.5)	

* is significant at the 0.05% level. ** is significant at the 0.01% level. *** is significant at the 0.001% level.

Finally, the results of the identification of factors affecting suicide attempts of those with mental health problems by logistic regression showed that at the confidence level of 73.0%, that the probability of completing a suicide was predicted by demographic factors and history of alcohol and substance use. The Omnibus test showed that the fit of the logistic model was acceptable. The values of the coefficient of determination (R²) showed that the presence of predictive factors could explain 8.0–11.0% of the changes in the variable of the probability of suicide completion. After running the regression model, the values of the Wald statistic and significance level in the logistic model showed that the presence of each of the factors of having a history of alcohol consumption (B = 0.375, $T_{wald}$ = 5.85, P = 0.016), the lower current age of the patient (B = −0.018,$T_{wald}$ = 4.50, P = 0.034), self-employed (B = −0.380, $T_{wald}$ = 4.597, P = 0.032), married (B = −0.746, $T_{wald}$ = 9.946, P = 0.002), younger at the first occasion of substance use (B = −0.026, $T_{wald}$ = 4.767, P = 0.029) and history of simultaneous substance use (B = 0.363, $T_{wald}$ = 3.915, P = 0.048) were all significant predictors in the logistic regression model (all PS < 0.05) for suicide attempts. The probability of committing suicide in those with a history of alcohol consumption was 1.5 times compared to those without a history of alcohol consumption, and 1.4 times in those with a history of simultaneous substance use compared to those without a history of simultaneous substance use. See Table 5 for a display of the regression model.

**Table 5:** Results of predicting suicide attempts based on demographic information and history of alcohol and substance use by the logistic regression model.

		B	S.E	Wald (Sig)	OR	95% C.I for Exp(B)	
						Lower	Upper
Constant		0.940	0.758	1.538 (0.215)	2.559	----	-----
**Factors**	The current age of the patient	−0.018	0.009	4.500* (0.034)	0.982	0.965	0.999
	Illiterate	0.325	0.470	0.478 (0.489)	1.384	0.551	3.479
	Primary school	0.313	0.377	0.687 (0.407)	1.367	0.653	2.864
	Elementary school	0.303	0.344	0.777 (0.378)	1.354	0.690	2.656
	High school	0.470	0.344	1.867 (0.172)	1.600	0.815	3.140
	University	0.539	0.481	1.257 (0.262)	1.715	0.668	4.401
	Student	−0.007	0.866	0.000 (0.993)	0.993	0.182	5.420
	Self-employed	−0.380	0.177	4.597* (0.032)	0.684	0.483	0.968
	Employee	0.013	0.341	0.001 (0.969)	1.013	0.520	1.975
	Single	−0.212	0.229	0.855 (0.355)	0.809	0.517	1.267
	Married	−0.746	0.236	9.946** (0.002)	0.474	0.298	0.754
	Female	0.399	0.343	1.351 (0.245)	1.490	0.761	2.919
	Father’s house	−0.864	0.537	2.589 (0.108)	0.421	0.147	1.207
	Rented house	−0.645	0.556	1.349 (0.245)	0.525	0.177	1.558
	Private house	−1.110	0.568	3.812 (0.051)	0.330	0.108	1.004
	First age of substance use	−0.026	0.012	4.767* (0.029)	0.974	0.951	0.997
	History of simultaneous substance use	0.363	0.184	3.915* (0.048)	1.438	1.003	2.061
	History of alcohol consumption	0.375	0.155	5.850* (0.016)	1.456	1.074	1.973

*significant at the level of 0.05%. **significant at the level of 0.01%

## Discussion

The present study was conducted with the aim of examining alcohol consumption as a risk factor for suicide among individuals with alcohol and substance use disorders (SUDs) in a cross-sectional study of the addiction registry in Kermanshah (western Iran). A number of demographic and substance use characteristics were surveyed and analyses examined which factors were the most significant predictors of suicidal ideation, attempts and mortality. The results revealed that the prevalence of suicide attempts was higher in men. In line with this finding, Zhang et al. [6]; Cao et al. [20] and Haaseet al. [21] also demonstrated that people who attempted suicide were more likely to be men. In the study by Cao et al. [20], the estimated ratio of males to females for lifetime prevalence of suicidal ideation and suicide attempts was 2.2 compared with 1.7, respectively. In the present study, suicide attempts were more common in people who had self-employment, young age, and unmarried people. The role of unemployment is important in increasing the vulnerability of individuals, especially young people, who make up the largest suicidal group. Findings indicated that job security had a great impact on preventing suicide attempts [22]. Unemployment contributes to negative emotions such as anxiety, anger, frustration and depression, which is likely to pose are risk factors for suicidal behavior [23].

It is likely financial strain and mental illness mutually reinforce one another. People with underlying psychiatric conditions are more likely to experience job loss or other financial hardship [24] and psychiatric disorders can impede recovery from economic stress [25]. Conversely, lower socioeconomic status may impede access to preventative mental health care and affect their ability to make decisions to optimize their health, leading commentators to conclude ―financial strain can be both a cause and a consequence of poor health [26]. These results were consistent with findings which showed that among male gender, low education level, unemployment, and being a housewife were more likely to have tried suicide attempts [6,23,27,28], but inconsistent with the results of studies Tavares et al. [29]; Caballero-Domínguez and Campo-Arias [30] and Koh et al. [31]. The discrepancies in the results may be due to differences in sample size, research tools, or cultural and social differences.

Also in line with previous findings [32–35], the present study findings demonstrate that alcohol consumption, among those with substance use disorders, was highly associated with suicidal ideation, suicide attempts, and suicide completions. Our analyses revealed that the probability of committing suicide among individuals seeking treatment for their substance use with a history of alcohol consumption was 1.5 times compared to those without a history of alcohol consumption. The results of the data analysis showed a significant statistical relationship between alcohol consumption and a history of suicide attempts, with a prevalence rate of 33.8% among individuals with a history of alcohol consumption. This finding is consistent with Vijayakumara, Kumarb, and Vijayakumar’s study [32] where they stated that 15% to 61% of individuals who attempted suicide had alcohol use disorders. Psychological data from China and Poland also showed similar rates of alcohol use disorders among suicide victims (15% and 17%, respectively). In comparison to these studies, the prevalence in Iran is higher, which can be attributed to cultural and social differences in alcohol consumption in the population. According to social epidemiological theories, the likelihood of suicide in an individual depends not only on their personal experiences but also on the mutual influence of cultural, economic, social, and environmental factors [33,34]. In line with the findings of this study, Richards et al. [35] stated in their study that patients who had high levels of alcohol consumption and suicidal ideation were ten times more likely to commit suicide compared to those with low alcohol consumption.

Recent studies of this association indicate that there are likely potential causal mechanisms between alcohol use disorders and suicidal behavior, even after accounting for genetic and familial environmental confounding [36,37]. It is not clear, however, if increasingly harmful alcohol consumption and alcohol-related behaviors across the general population show proportionate associations with suicidal behavior [38]. Also, in explaining this finding, it has been suggested that the experience of negative life events along with untreated psychiatric disorders in alcohol users can push them toward suicide [39]. On the other hand, the increased accessibility to lethal substances, legal and occupational problems, and loss of close relationships following alcohol misuse and dependence can be possible reasons for the higher suicide rate in this group of individuals [40]. Additionally, acute alcohol consumption may reduce individuals’ tolerance threshold for coping with life challenges and decrease their ability to tolerate distress, putting them at risk of suicidal thoughts or actions. Moreover, individuals who are regular alcohol users may exacerbate negative mood states such as anxiety and aggression, promoting suicidal behavior in them. Future research is needed to understand the relationship among these variables [41]. Additionally, based on physiological theoretical foundations, acute alcohol intoxication can increase impulsivity and impair inhibition, judgment, and problem-solving abilities, making individuals more vulnerable to suicidal thoughts or related behaviors [42], but psychophysiological studies would be needed to further investigate this area.

In the present sample, the probability of committing suicide in those with a history of simultaneous substance use was 1.4 times more compared to those without simultaneous substance use. In their study, they also stated that 33% of people below the age of 20 who harmed themselves had a high level of alcohol and substance use. In some studies, it has been estimated that 25–50% of all suicides are due to substance use and 22% are related to alcohol consumption [8]. The risk of suicidal thoughts will increase significantly among substance users [43]. In a study by Wines et al. [44], on a group of substance users, suicidal thoughts were 28.5% in the year before treatment, and 10.4–19.9% even after treatment (2-year follow-up). Examinations of outpatient versus inpatient populations Driessen et al. [45]; Darvishi et al. [46], demonstrated that substance use is the second most common reason (22.4%) for completed suicides in outpatient settings, which was twice that in inpatient settings. A meta-analysis of studies of risk factors for suicidal deaths identified that mood disorders and SUDs increased the risk of suicide 7–9 times [47]. SUDs were significant predictors of suicide attempts in the days and months before committing suicide [48]. SUDs are associated with significant risk of suicide mortality. These findings suggest increased suicide risk screening and prevention efforts for individuals with substance use disorders are needed.

The present study has limitations, including the reliance on interview-based data and the lack of control over predictors of suicide in hospitalized substance-dependent individuals, such as socio-economic factors. In addition, the prevalence of suicide attempts may be higher among substance abusers, but due to the lack of access to participants’ medical records, we cannot be certain about the accuracy of their reports. Furthermore, some participants may have a history of suicidal thoughts or attempts but may have refrained from mentioning them due to social stigma. This issue can be addressed by normalizing the disclosure of suicidal thoughts during interviews, combined with active listening and providing a caring environment by interviewers, without unnecessary alarm or distress. Moreover, based on the research data, it is not possible to judge the timing of suicide attempts by participants (before or after the onset of addiction) since reliable information in this regard was not collected.

In the future research, it is suggested to investigate the prevalence of suicidal ideation and attempts and identify their risk factors in people with substance use disorder. Also, future studies can address the limitations of our cross sectional design through longitudinally examining the predictive role of protective factors against suicide. In the assessment and psychological interventions for substance users participating in addiction treatment programs, consideration should be given to a history of suicide attempts and factors that contribute to their prevention, especially taking into account a history of alcohol consumption. Considering these limitations, the results cannot be generalized beyond our study population, individuals receiving inpatient care for their substance use disorders in Iran.

## Conclusion

In general, based on the findings of the research, Iranian individuals with co-occurring substance use disorder and alcohol consumption should be considered a high-risk group for suicidal behavior. Therefore, it is advisable to incorporate suicide screening as a regular part of the clinical assessment and treatment of substance and alcohol users. Similarly, regular screening for suicide ideation in alcohol treatment settings may be a useful strategy for suicide prevention. The risk of suicide attempts is higher in individuals who simultaneously use substances and alcohol, thus controlling and treating substance and alcohol abuse can contribute to the prevention of suicide attempts and suicidal ideation. The findings of this research can inform the provision of treatment and care for these Iranian individuals during hospitalization and be made available to nurses, physicians, and all those who interact with these individuals in any capacity.
